# Intestinal Morphometric Changes Induced by a Western-Style Diet in Wistar Rats and GSPE Counter-Regulatory Effect

**DOI:** 10.3390/nu14132608

**Published:** 2022-06-23

**Authors:** Helena Segú, Florijan Jalševac, Montserrat Pinent, Anna Ardévol, Ximena Terra, Maria Teresa Blay

**Affiliations:** Mobiofood Research Group, Departament de Bioquímica i Biotecnologia, Universitat Rovira i Virgili, Campus Sescelades, C/ Marcel·lí Domingo 1, 43007 Tarragona, Spain; helena.segu@urv.cat (H.S.); florijan.jalsevac@urv.cat (F.J.); montserrat.pinent@urv.cat (M.P.); anna.ardevol@urv.cat (A.A.); mteresa.blay@urv.cat (M.T.B.)

**Keywords:** western-style diet, intestine morphology, absorptive surface area, Wistar rats, proanthocyanidins

## Abstract

Western-style diet is an obesogenic diet for rodents and humans due to its content of saturated fat and refined sugars, mainly sucrose and, in consequence, sucrose-derived fructose. This type of diets relates with intestinal disturbances when consumed regularly. The aim of this work was to analyse the adaptive morphologic and functional changes at intestinal level derived from the unhealthy components of a Cafeteria diet in rats. The effect of grape seed proanthocyanidin extract (GSPE) in the prevention of diet-induced intestinal dysfunction was also analysed. Rats were fed a 17-week cafeteria diet (CAF) without or with oral-GSPE supplementation, either intermittent GSPE administration (SIT-CAF); last 10-day GSPE supplementation at doses of 100 mg/kg and 500 mg/kg day (CORR-100) and (CORR-500) or pre-supplementation with 500 mg/kg GSPE (PRE-CAF). GSPE-CAF supplemented groups showed similar results to CAF diet group regarding morphology and inflammatory score in the duodenum. As an adaptive response to diet, CAF increased intestinal absorptive surface (1.24-fold) all along the intestinal tract and specifically in the small intestine, duodenum, due to increase villus height and a higher villus/crypt ratio, in addition to increase in Goblet cell percentage and inflammatory index. Animals fed GSPE at the current doses and times had higher villus heights and absorptive surface similar to Cafeteria diet group. In the duodenum, villus height correlated with body weight at 17 week and negatively with MLCK gene expression. In the colon, villus height correlated with the percentage of goblet cells. In conclusion, the CAF diet produced adaptive modifications of the intestine by increasing the absorptive area of the small intestine, the percentage of goblet cells and the inflammatory index at the duodenal level. GSPE supplementation can partially reverse the intestinal morphological changes induced by the high fat/sucrose diet when administered intermittently.

## 1. Introduction

The Western diet characterises by the high consumption of energy-dense ultra-processed foods, rich in saturated fats and simple carbohydrates. In this dietary pattern, more than 14% of the calorie intake comes from sugars, with sweet products and sweetened beverages as the main contributors to sugar consumption [1,2]. As a result, concerns have raised regarding the impact of these food products on the global epidemic of obesity [3]. The World Health Organization recommends limiting added sugars to one-tenth of the daily calorie intake, namely 50 g per day for a 2500-calorie diet as well as European Food Safety Authority (EFSA) [4]. Nevertheless, a typical 330-mL can of a regular soft drink contains up to 40 g of free sugars (>600 mmol L^−1^). In addition, most foods marketed as healthy choices contains large portions of sugar (e.g., some breakfast cereals, yogurt, and other sugar-sweetened dairy products) and daily intake per capita in some developed countries can reach up to 145 g [1].

As we reported in previous articles, rats fed a Western-style cafeteria diet (rich in sucrose and saturated fats) not only gain body weight and increased organ weights (liver, white adipose tissue, thymus, kidney, brown adipose tissue) but also manifest mild intestinal inflammation and enhanced permeability (intestinal dysfunction) [5,6,7].

Proanthocyanidins are a group of flavonoids present in a variety of botanical sources such as cocoa, nuts, fruit and spices, which have demonstrated among others, antioxidant, anti-inflammatory and immunomodulatory effects in vivo [8,9], and could offer a safe adjunctive support to prevent the deleterious effects of unbalanced diet (“junk diet”), overweight and obesity [10,11,12].

Oral administration of grape seed proanthocyanidins extract (GSPE) to rats has demonstrated beneficial effects against the increased intestinal permeability, inflammation and body weight developed because of a long-term cafeteria diet. We previously demonstrated that an intermittent GSPE treatment for 5 weeks during the administration of cafeteria diet normalized intestinal permeability and inflammation to levels comparable to the standard diet group up to 17 weeks in contrast to the non-GSPE cafeteria fed group, which maintained increased intestinal permeability and inflammation. The myeloperoxidase (MPO) activity, a marker of intestine’s inflammation, increased in the cafeteria diet group and decreased in preventive GSPE-receiving group [5,7].

The histochemical parameters of the gastrointestinal tract of rats have been studied previously by Sharma et al. [13]. However, the effect of Cafeteria diet on intestinal morphology has been partially described and the effect of GSPE supplementation together with cafeteria diet in Wistar rats has not been studied.

In this study, we aimed to investigate the effect of cafeteria diet with or without a GSPE preventive treatment in the morphology and cell composition of small and large intestines. In addition, what would be the contribution of GSPE to putative morphological changes in the context of the typical Western diet? Does GSPE promotes alterations in the morphology of intestine? How any alteration correlates with functional/gene expression parameters in small and large intestine and in the whole body is addressed again.

## 2. Materials and Methods

### 2.1. Proanthocyanidin Extract

The grape-seed proanthocyanidin extract (GSPE) was provided by Les Dérivés Résiniques et Terpéniques (batch number 124029; Dax, France). According to the manufacturer, the composition of the GSPE is as follows: monomers of flavan-3-ols (21.3%), dimers (17.4%), trimers (16.3%), tetramers (13.3%) and oligomers (5–13 units; 31.7%) of proanthocyanidins. A detailed analysis of the monomeric, dimeric, and trimeric structures of the GSPE can be found in the work of Margalef et al. [14], who studied the main phenolic compounds of this extract by HPLC-MS/MS. The GSPE was dissolved in ethanol absolute to prepare a stock solution of 50 mg mL^−1^.

### 2.2. Animal Models

Forty-seven-week-old female Wistar rats (240–270 g) purchased from Charles River Laboratories (Barcelona, Spain) were individually caged in animal quarters at 22 °C with a 12-h light/12-h dark cycle and fed a standard (STD) chow diet (Panlab 04, Barcelona, Spain) and tap water *ad libitum*. After an acclimation period, animals were randomly distributed into six experimental groups (*n* = 10). The control group (STD group) received only the STD diet. The other five groups were fed a cafeteria (CAF) diet as a model of high saturated fat/high sucrose diet until the end of the animal experiment. The CAF diet was offered *ad libitum* and replenished every day with a quantity that was enough for 17 weeks. CAF-fed animals also had free access to standard chow. The composition of the diets supplied is shown in Table 1. One of the groups of the CAF diet also received a daily preventive treatment of GSPE (PRE-CAF), 10 days before the long-term cafeteria intervention, at a dose of 500 mg of GSPE/kg of body weight (bw) per day. Another group received the CAF diet simultaneously with daily proanthocyanidins treatment (500 mg GSPE/kg day) every other week (Simultaneous-Intermittent-Treatment-CAF; SIT-CAF) for the 17 weeks. During the final two weeks of the CAF intervention, another two CAF-diet groups received daily doses of 100 and 500 mg GSPE/kg of bw as a corrective treatment (groups CORR100 and CORR500, respectively).

**Figure 1:**
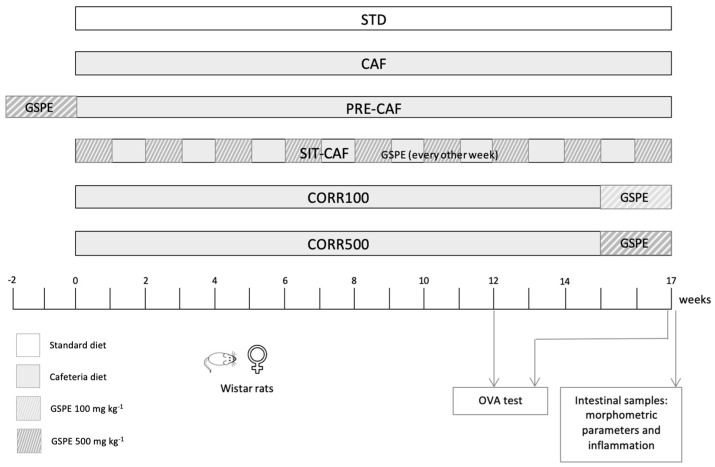
Experimental design. Schematic diagram of the animal experimental design. STD, rats fed a standard chow diet; CAF, rats fed a cafeteria diet; PRE-CAF, rats fed a cafeteria diet plus a preventive treatment with 500 mg/kg GSPE; SIT-CAF, rats fed a cafeteria diet with the GSPE treatment every other week; CORR100 and CORR500, rats fed a cafeteria diet plus a corrective treatment with 100 or 500 mg/kg GSPE.

The GSPE was dissolved in water and orally gavaged every day to each animal at 18:00 hour in a final volume of 0.5 mL. Non-supplemented animals received water as a vehicle. The experimental design is shown in Figure 1. All procedures involving the care and use of animals in this work were reviewed and approved by Animal Ethics Committee at Universitat Rovira i Virgili (code: 0152S/4655/2015).

### 2.3. Histological Analysis of Intestinal Sections and Histopathology Inflammatory Scoring

Intestinal length of the intestine was measured after excision with a ruler as a measure of the effect of diet intervention to animals with a millimeter ruler.

Small and large intestinal samples from each animal and intestinal section (duodenum, ileum and colon) were fixed in a 4% formaldehyde solution for 24 h, transferred to a 70% ethanol solution until paraffin inclusion. Tissue sections 4 µm thick were cut from paraffin blocks and placed on glass slides.

Haematoxylin and eosin (H&E) staining was performed using standard procedures from sections from the small intestine (duodenum and ileum) and large intestine (colon). Three sections of each intestinal section were determined. We used 3–8 animals from each experimental group.

Under a light microscope, tissue architecture, epithelium morphology and the percentage of Goblet cells were analysed in the preparations of duodenum, ileum and colon segments at Eldine Laboratories (Tarragona, Spain). The selected slides (three per animal and intestinal location) were reviewed without prior knowledge of the animal’s experimental group. Crypt width data is estimated.

**Figure 2:**
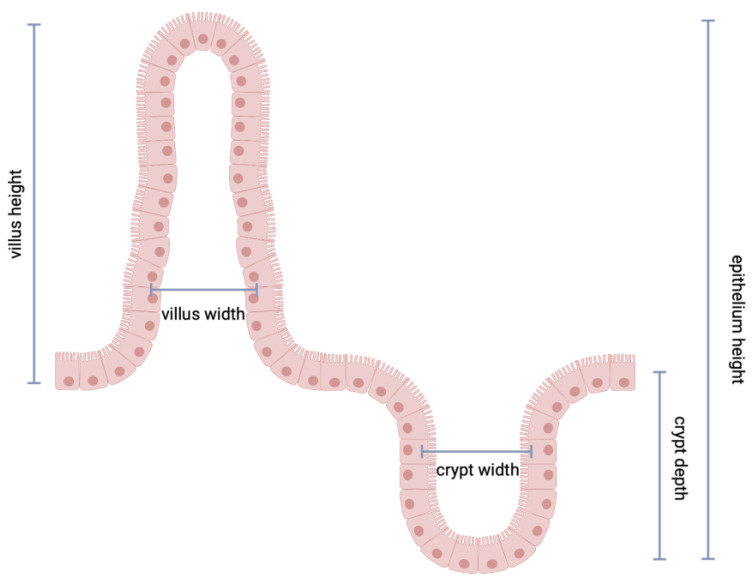
Parameters measured in the duodenum, ileum and colon samples.

The following parameters were measured: (1) villus height (distance from the villus-crypt junction to the top of the villus); (2) villus width (measured at the villus, distance between the villus-crypt junctions); (3) crypt depth (depth of the invagination between adjacent villi); (4) crypt width (measured at the crypt, distance between the villus-crypt junctions) total epithelium height (distance from the top of the villus and the bottom of the crypt); (5) villus/crypt ratio (see in Figure 2). To quantify the absorptive area of each intestinal fragment, we followed the protocol of Kisielinski et al. [15], which describes an equation that includes villus height, villus width and crypt depth.

For inflammatory status assessment, related histological changes were on a scale of 0–3. The inflammatory index was measured as chronic index or an acute index (0–3) indicating: absence of inflammation-grade = 0, low-grade inflammation-grade 1, intense inflammation-grade = 2, severe inflammation-grade = 3 by immune cell infiltration of mature and immature forms assessment under the optical microscope by a pathologist (see Table 2). The distribution of the mature forms was also evaluated.

### 2.4. Statistical Analysis

Results were expressed as the mean ± SEM. Statistical comparisons between groups were assessed by *t*-test. *p*-values < 0.05 were considered statistically significant. Analyses were performed with the XLSTAT Premium 2022 Module (Addinsoft, New York, NY, USA). Pearson coefficients were also calculated with XLSTAT Premium 2022 software.

## 3. Results

### 3.1. Cafeteria Diet Modified Epithelium Morphology In Vivo

The lengths of small and large intestines as well as the total intestinal length unchanged by diet or proanthocyanidin treatment (see Table 3).

**Figure 3:**
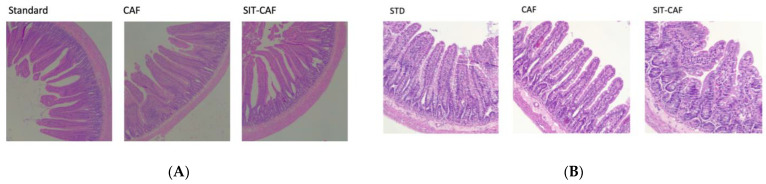
(**A**) Haematoxylin and eosin (H&E) staining of duodenum samples of STD, CAF, and SIT-CAF groups. CAF, rats fed a cafeteria diet; STD, rats fed a standard chow diet; (**B**) Haematoxylin and eosin (H&E) staining of ileum samples of STD, CAF, and SIT-CAF groups. CAF, rats fed a cafeteria diet; STD, rats fed a standard chow diet; SIT, simultaneously intermittent GSPE treatment (500 mg/kg body weight).

Haematoxylin and eosin (H&E) staining of the duodenum, ileum, and colon (see Figure 3) and optical assessment allowed measuring the morphometric intestinal variables. The administration of a cafeteria diet for 17 weeks to Wistar rats significantly increased the villous height, the M ratio of absorptive surface and the villus/crypt ratio of the duodenum and epithelium width with respect to animals that ingested standard chow diet.

The preventive GSPE administration of 500 mg of GSPE/kg of body weight did not modify any morphologic intestinal parameter and were similar to cafeteria diet animals, but different to standard diet ones (see Table 4, Table 5 and Table 6). The intermittent treatment of GSPE did normalize the duodenal villus/crypt ratio, M factor and villus and epithelium height to standard values.

Moreover, no changes of morphological intestinal parameters in the obesogenic diet rats were observed in other GSPE treatments of corrective 10-day intervention at the end of cafeteria diet intake at 100 or 500 mg GSPE/kg body weight.

The inflammatory index measured as chronic index or acute index independently did not show significant differences in the duodenum by effect of CAF diet.

When the acute and chronic inflammatory parameters (chronic inflammatory index and acute inflammatory index) were analysed in standard diet, cafeteria diet and GSPE-CAF diet supplemented groups and analysed together we could find in the duodenum samples that the cafeteria diet induced a higher level of inflammation (Table 6). In the SIT-CAF treatment, no differences were found when compared to CAF group.

### 3.2. Cafeteria Diet Did Not Modify Goblet Cell Content

To evaluate the influence of Cafeteria diet and GSPE-supplementation on intestinal cell composition, we analysed the percentage of Goblet cells in Haematoxylin and Eosin preparations of duodenum, ileum and colon segments.

The cafeteria diet showed a significant change of Goblet cell percentage in these intestinal fragments (see Table 7). Moreover, the percentage of Goblet cells was significantly higher in the colon (large intestine) than in the duodenum (small intestine) in the control diet and cafeteria diet groups (*n* = 6–8).

The composition of the Goblet cells did exert an increase in Cafeteria diet group and PRE-CAF group and a decrease or a tendency to decrease between the GSPE-supplementation groups (SIT-CAF, CORR100 and CORR500).

### 3.3. Correlations of Morphological Parameters and Metabolic Parameters in Wistar Rats 

To evaluate the possible correlation of morphological parameters in the intestine and functional characteristics, we analysed the correlations in duodenum, ileum and colon segments between villus height and crypt depth and functional parameters as body weight, gene expression, and percentage of Goblet cells.

We found a positive significant correlation (Spearman correlation r, p) between the villus height in the duodenum with final body weight of rats and a negative correlation with myosin light chain kinase (MLCK) gene expression. In colon, we find a positive correlation between crypt depth and the percentage of Goblet cells. There was no correlation of Villus height in the duodenum, ileum and colon and proinflammatory plasma cytokine levels (IL-1β, TNF-α) or inflammation- related gene expression of (Emr1, IL-13, IL-1β, TNF-α) or MPO activity. There was also no correlation between villus height and tight junction’s gene expression Ocludin-1 and Claudins-1 and 2. No correlation was found in the ileum (Table 8).

## 4. Discussion

It is widely known that the Western diet induce changes in the gastrointestinal tract functionality. Published articles from authors and from our research group report alterations of intestinal permeability and inflammation in rats fed with a high fat/refined carbohydrate diet [6,7,16,17]. On this basis, first, our aim in the present study was to assess the structural responses of small and large intestine of Wistar rats to cafeteria diet rich in added sucrose (and sucrose-derived fructose) and to GSPE supplementation.

This study shows that chronic ingestion of cafeteria diet exerts an increase in the height of rat duodenal villi and total epithelium, and in the villus/crypt ratio, contributing to the overall absorption area or M surface area amplification. We suggest that this is a dietary adaptation to increase the caloric availability of food intake, as has been observed in other publications linking dietary macronutrients consumption to morphological changes in the intestine.

It is known that Wistar rats respond to protein content by modulating the dimensions of the villus in the small intestine [18]. We have administered a cafeteria rich diet that enhances the intake of refined carbohydrates and fat in detriment to protein content. However, we also find this adaptive response in the increase in villus height, which depends on the continuous renewal of the intestinal epithelium through the multiplication and differentiation of stem cells at the base of the crypt, and the migration of mature cells along the villus [18,19].

In the present study, the main source of carbohydrate intake was sucrose, which was taken daily at a rate of 46 g per 100 g of diet. A daily intake of a minimum of 100 mL of milk with sugar was also recorded, which means that sucrose was the main source of energy followed by fat. Sucrose is broken down into fructose and glucose, which are postulated to be the main agents causing this adaptive response [20], increasing the surface area to optimize and enhance the absorption of these components [21]. In this regard, Taylor et al. reported a 25–40% villus length increase in the duodenum and proximal jejunum in mice fed with high-fructose corn syrup compared to control ones [22]. All this evidence together with our results suggest that not only protein content is able to affect and determine intestinal morphology, but also carbohydrates. In addition, fat intake and the overall caloric content of the diet have also been reported to influence intestinal morphology by increasing absorption parameters [23].

While in the small intestine we could observe an increase in the intestinal absorption surface which is adapted to absorb the maximum amount of energy in the cafeteria group (junk diet); in the colon, where the maximum absorption of water but not nutrients occur, this adaptation is not found.

Regarding cell content adaptation in the intestine, it is seen that goblet cell content increases along the intestinal tract of Wistar rats and that cafeteria diet causes an increase in goblet cell content in the duodenum. Goblet cells are specialized in the synthesis and production of mucus that grows from the lower to the upper gastrointestinal tract [19], as we observed in the present study.

With respect to the presence of mature and immature inflammatory forms in the duodenum analysed by expert histopathologists, we found a basal level of inflammation in standard diet consuming animals and a significant increase of inflammatory cells infiltration (rate of total inflammation accumulated) when rats consumed cafeteria diet.

Cafeteria diet also influence intestinal barrier function. Gene expression analysis of barrier related proteins showed a negative correlation between myosine light chain kinase (MLCK) and villus height in the duodenum. The biological meaning of this negative correlation between MLCK gene expression and villus height in this context is difficult to explain. Paracellular permeability is principally determined by the phosphorylation level of the regulatory light chain of myosin-2 (MLC-2), which is regulated by the enzyme MLCK. Regulation of MLC-2 phosphorylation by MLCK leads to contraction of the actin skeleton thereby increasing the permeability of the paracellular barrier in intestinal epithelial cells [24]. In our work, the groups with higher villus height also showed higher intestinal permeability, which is a paradox. However, with intestinal permeability established after 17 weeks of junk diet, the effect of MLCK at the expression level is not as relevant as its activity.

On the other hand, we also found a positive correlation between final body weight gain and duodenal villus height and a positive correlation between villus height and food intake. Previous results from our group showed a positive correlation between body weight gain and food calorie intake in Wistar rats on a standard or cafeteria diet, and also demonstrated that there was a modulation of food intake by GSPE treatment [6,17].

Grape seed proanthocyanidin extract called GSPE is a natural, non-nutritive compound that is added to the diet. In the present study, we wanted to see if GSPE had any effect on modulating gut morphology. We believe that the fact that GSPE did not modify intestinal morphology at the concentrations tested in the corrective treatment of 100 and 500 mg/kg/day (low and high) or in the preventive supplementation is indicative of the absence of deleterious effects of the extract.

However, the SIT treatment with proanthocyanidins was able to reduce and normalize villus-to-crypt ratio, the villus and epithelium heights and M-ratio, indicating that this treatment is optimal for animals fed an unbalanced cafeteria diet. As well as grape seed-proanthocyanidin extract derived from *Vitis vinifera*, other plant-derived compounds such as sapogenin extract of Jamaican bitter yam (*Dioscorea polygonoides*) are able to modify the structure of intestine thus ameliorating deleterious effects of junk diets [25].

GSPE extract also normalised the goblet cell content of the duodenum in the intermittent and corrective treatments to levels comparable to those of animals fed standard diet, only with significance in the CORR500 group. The preventive treatment was not able to prevent the increase in goblet cell numbers caused by the cafeteria intervention. Thus, the reduction in goblet cell numbers with GSPE treatments compared to the CAF group only occurred in the duodenum, but not in the ileum and colon.

Regarding diet-associated inflammation, chronic GSPE did not change the index of acute, chronic or total inflammation in the duodenum; but, as previously reported, GSPE treatments decrease MPO activity, an indicator of neutrophil infiltration, in the same animals [17,26].

In summary, the sucrose-rich cafeteria diet chronically administered to rodents caused morphological changes in the intestine by increasing the surface area of intestinal absorption, probably due to the glucose and fructose content following sucrase action, as an adaptative response. Treatment with 500 mg GSPE/kg.day intermittently every two weeks was the optimal dose-time of supplementation, as it prevented the increase in intestinal absorption surface area induced by chronic ingestion of the cafeteria diet.

## Figures and Tables

**Table 1:** The composition and energy content of diets administered.

	STD	CAF
**Content (g 100 g** **^–1^** **dry matter)**		
Available carbohydrate	48.0	62.6
Sugar (sucrose)	≈0.0	46.0
Protein	14.3	15.1
Fat	4.0	17.2
Saturated	0.6	8.1
Fibre	4.1	1.7
**Energy contribution**		
kJ g^–1^ dry matter	12.1	20.7
Carbohydrate (%)	67.2	52.0
Protein (%)	20.2	14.1
Fat (%)	12.6	33.9

STD diet, standard chow diet; CAF diet, cafeteria diet. The CAF diet consisted of bacon, sausages, biscuits, pateé, carrots, muffins, and sugared (sucrose) milk, which induces voluntary hyperphagia.

**Table 2:** Scoring criteria of measured inflammation parameters.

Scoring Criteria		Score
Chronic inflammation (IC) = number of mature forms	Absent	0
	Slight	1
	Moderate	2
	Intense	3
Distribution of IC	Diffuse	
	Aggregate	
	Diffuse and Aggregate	
Acute inflammation (IA) = number of immature forms	Absent	0
	Slight	1
	Moderate	2
	Intense	3

**Table 3:** Intestinal length in experimental groups at 17 weeks. STD group, rats fed standard chow diet; CAF group, rats fed cafeteria (CAF) diet; CAF-fed rats supplemented with a 500 mg kg^−1^ b w of GSPE. The number of animals included in this study was *n* = 10 for each group. Values indicate mean ± standard error (SEM).

Morphometric Parameters	STD	CAF	SIT-CAF
Small intestine length (cm)	101.78 ± 2.1	105.3 ± 2.1	104.5 ± 1.3
Colon length (cm)	16.2 ± 0.9	15.4 ± 0.4	17 ± 0.6
Total length (cm)	117.8 ± 2.9	120.8 ± 2.3	119.6 ± 2.6

**Table 4:** Morphometric intestinal variables affected by cafeteria diet and a GSPE treatment in Wistar rats. SIT, simultaneously intermittent GSPE treatment (500 mg/kg body weight). Values represent mean ± SEM; *n* = 10. In bold most relevant changes.

Small Intestine	STD	CAF	SIT-CAF
Duodenum			
**Villus height (µm)**	610.39 ± 22.07	734.67 ± 16.45 *	657.38 ± 27.17
Villus width (µm)	142.74 ± 10.83	160.90 ± 7.78	126.28 ± 18.26
Crypt depth (µm)	189.02 ± 8.63	196.92 ± 6.69	197.56 ± 8.42
Crypt width (µm)	48.87 ± 1.47	45.90 ± 1.27	47.10 ± 1.28
**Epithelium height (µm)**	806.41 ± 22.22	939.46 ± 20.59 *	864.84 ± 33.72
**Villus/crypt ratio**	2.95 ± 0.11	3.77 ± 0.09 *	3.28 ± 0.38
**M (surface area amplification ratio)**	9.56 ± 0.83	11.87 ± 0.56*	11.04 ± 0.43
Ileum			
Villus height (µm)	229.33 ± 16.78	242.29 ± 21.15	242.53 ± 10.95
Villus width (µm)	136.49 ± 21.78	121.12 ± 13.35	119.71 ± 11.88
Crypt depth (µm)	124.91 ± 9.45	127.62 ± 12.19	131.10 ± 8.42
Crypt width (µm)	44.02 ± 3.36	42.45 ± 0.51	47.10 ± 1.28
Epithelium width (µm)	336.52 ± 20.18	369.91 ± 31.63	373.63 ± 16.52
Villus/crypt ratio	1.85 ± 0.08	1.91 ± 0.06	1.87 ± 0.10
**Large Intestine**	**STD**	**CAF**	**SIT-CAF**
Colon			
Crypt depth (µm)	155.24 ± 4.45	166.55 ± 7.61	156.36 ± 7.96

* indicate the statistically significant differences with respect to standard diet group (*p* < 0.05) by Student *t* test.

**Table 5:** Morphometric intestinal variables affected by cafeteria diet and GSPE treatments in Wistar rats. CORR100, corrective 10-day intervention at 100 mg/kg body weight; CORR500, corrective 10-day intervention at GSPE 500 mg/kg body weight. Values represent mean ± SEM; *n* = 10. In bold most relevant changes.

Small Intestine	PRE-CAF	CORR100	CORR500
**Duodenum**			
**Villus height (µm)**	766.39 ± 29.67 *	818.27 ± 31.36 *	726.66 ± 32.71 *
Villus width (µm)	147.28 ± 8.53	138.86 ± 17.43	146.42 ± 11.95
Crypt depth (µm)	189.83 ± 9.46	199.62 ± 6.06	194.71 ± 4.09
Crypt width (µm)	46.04 ± 0.67	45.17 ± 2.04	46.44 ± 1.17
**Epithelium width (µm)**	936.57 ± 21.55 *	1022.84 ± 27.66 *	928.32 ± 37.64 *
**Villus/crypt ratio**	3.98 ± 0.25 *	3.66 ± 0.17 *	3.62 ± 0.15 *
**M (surface area amplification ratio)**	12.55 ± 0.73 *	13.63 ± 1.38 *	12.17 ± 0.84 *
**Large Intestine**	**PRE-CAF**	**CORR100**	**CORR500**
**Colon**			
Crypt depth (µm)	151.88 ± 9.38	162.96 ± 9.81	169.66 ± 8.96

* indicate the statistically significant differences with respect to standard diet group (*p* < 0.05) by Student *t* test.

**Table 6:** Inflammatory parameters affected by cafeteria diet and GSPE treatments on intestinal index of inflammation in Wistar rats. SIT, simultaneously intermittent GSPE treatment (500 mg/kg body weight). Values represent mean ± SEM; *n* = 3–8.

Small Intestine	STD	CAF	SIT-CAF
Duodenum	Grade	Grade	Grade
Index of chronic inflammation	0.56 ± 0.24	0.67 ± 0.17	1.00 ± 0.00
Index of acute inflammation	0.83 ± 0.11	1.33 ± 0.33	1.00 ± 0.00
Inflammatory index	1.39 ± 0.17	2.00 ± 0.25 *	2.00 ± 0.00 *
(accrued)			

* indicate the statistically significant differences with respect to standard diet group diet group (*p* < 0.05) by Student *t* test.

**Table 7:** Percentage of Goblet cells in duodenum, ileum and colon H&E slides in different groups. Values represent mean ± SEM; *n* = 3–8 animals.

% Goblet Cells	STD	CAF	PRE-CAF	SIT-CAF	CORR100	CORR500
**Duodenum**	9.13 ± 1.02	12.17 ± 1.11 *	11.26 ± 0.7 *	10.83 ± 0.87	10.00 ± 1.41	9.75 ± 0.45 ^#^
**Ileum**	21.43 ± 0.72	20.40 ± 1.89	n.a	20.20 ± 1.24	n.a.	n.a
**Colon**	58.00 ± 3.78	58.33 ± 4.36	58.90 ± 3.03	64.50 ± 3.80	60.71 ± 3.08	65.11 ± 2.69

* indicate the statistically significant differences with respect to standard diet group diet group (*p* < 0.05) by Student *t* test. # indicate statistically significant differences with respect to cafeteria diet group.

**Table 8:** Pearson correlation coefficients of intestine parameters in Wistar rats.

**Correlation Matrix Pearson**	**Villus Height-Duodenum**	**Crypt Depth Colon**
Final Accrued body weight gain (*n* = 24)	0.476	
Final food intake (*n* = 58)	0.368	
MLCK gene expression (*n* = 31)	−0.369	
% Goblet cells (*n* = 52)		0.379
